# Susceptibility to hippocampal kindling seizures is increased in aging C57 black mice

**DOI:** 10.1016/j.ibror.2017.08.001

**Published:** 2017-09-24

**Authors:** Kurt R. Stover, Stellar Lim, Terri-Lin Zhou, Paul M. Stafford, Jonathan Chow, Haoyuan Li, Nila Sivanenthiran, Sivakami Mylvaganam, Chiping Wu, Donald F. Weaver, James Eubanks, Liang Zhang

**Affiliations:** a Krembil Research Institute, University Health Network, Canada; b Departments of Chemistry, University of Toronto, Canada; c Departments of Medicine, University of Toronto, Canada; d Departments of Surgery, University of Toronto, Canada; e University of Toronto Epilepsy Program, Canada

**Keywords:** Aging, EEG, Epilepsy, Hippocampus, Kindling, Mice

## Abstract

The incidence of seizures increases with old age. Stroke, dementia and brain tumors are recognized risk factors for new-onset seizures in the aging populations and the incidence of these conditions also increased with age. Whether aging is associated with higher seizure susceptibility in the absence of the above pathologies remains unclear. We used classic kindling to explore this issue as the kindling model is highly reproducible and allows close monitoring of electrographic and motor seizure activities in individual animals. We kindled male young and aging mice (C57BL/6 strain, 2–3 and 18–22 months of age) via daily hippocampal CA3 stimulation and monitored seizure activity via video and electroencephalographic recordings. The aging mice needed fewer stimuli to evoke stage-5 motor seizures and exhibited longer hippocampal afterdischarges and more frequent hippocampal spikes relative to the young mice, but afterdischarge thresholds and cumulative afterdischarge durations to stage 5 motor seizures were not different between the two age groups. While hippocampal injury and structural alterations at cellular and micro-circuitry levels remain to be examined in the kindled mice, our present observations suggest that susceptibility to hippocampal CA3 kindling seizures is increased with aging in male C57 black mice.

## Highlights

• Hippocampal kindling by daily stimulation conducted in aging and young mice.
• Stimulation parameters were comparable in the two groups of mice.
• No significant age difference in cumulative afterdischarges to stage 3–5 seizures.
• Longer afterdischarges and faster seizure progression observed in the aging mice.
• Aging mice may have increased susceptibility to hippocampal kindling seizures.

## Graphical abstract

**Figure:**
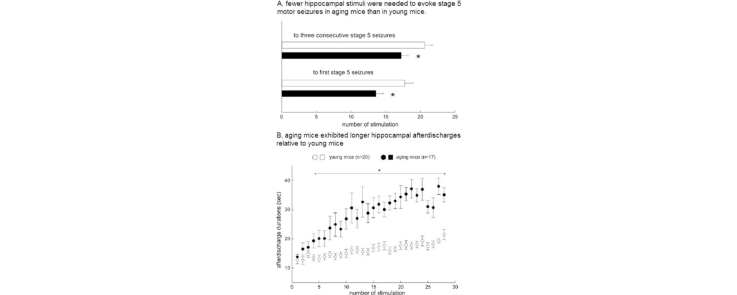

## Introduction

Old age is associated with high incidence of seizures and epilepsy, and temporal lobe epilepsy is the most common type of seizure disorder in aging/aged populations. While stroke, dementia and brain tumors are recognized risk factors, the etiology is unknown for many aging/aged individuals with new-onset epilepsy (Hauser, 1992, Jetter and Cavazos, 2008, Brodie et al., 2009, Ferlazzo et al., 2016). This raises an intriguing question as to whether intrinsic processes within the aging brain promote seizures/epilepsy susceptibility (Baram, 2012).

Experimental investigation using animal models may help explore this issue. However, studies that compare seizure susceptibility between young and aging/aged animals remain scarce (de Toledo-Morrell et al., 1984, Fanelli and McNamara, 1986, Leppik et al., 2006, Kelly, 2010, Hattiangady et al., 2011).

The hippocampus is known to undergo structural and functional alterations during aging (Burke and Barnes, 2010, Bartsch and Wulff, 2015). These include a loss of subgroups of hippocampal GABAergic interneurons (Shetty and Turner, 1998, Cadacio et al., 2003, Vela et al., 2003, Shi et al., 2004, Stanley and Shetty, 2004, Potier et al., 2006, Kuruba et al., 2011, Smith et al., 2000, Stanley et al., 2012, Spiegel et al., 2013) and an increase in hyperactive or hyperexcitable responses of hippocampal CA3 neurons in aging/aged animals (Vreugdenhil and Toescu, 2005, Wilson et al., 2005, Patrylo et al., 2007, Kanak et al., 2011, Lu et al., 2011, El-Hayek et al., 2013, Spiegel et al., 2013, Moradi-Chameh et al., 2014, Simkin et al., 2015, Villanueva-Castillo et al., 2017). In light of these findings and classic kindling as a widely used model of temporal lobe epilepsy (see reviews by Morimoto et al., 2004, Bertram, 2007, Sharma et al., 2007, Coppola and Moshé, 2012, Gilby and O'Brien, 2013, Chauvette et al., 2016, Gorter et al., 2016, Löscher, 2017), we explored whether susceptibility to hippocampal CA3 kindling seizures is different between young and aging C57 black mice.

## Experimental procedures

### Animals

Male C57 black mice (C57BL/6N) were obtained from Charles River Laboratory (Senneville, Quebec, Canada). Experiments were carried out on mice of 2–3 and 18-22 months of age. C57 black mice have a maximum lifespan up to 36–39 months (Flurkey et al., 2007, Harrison et al., 2009) but mice of ≥24 months of age often encounter health-related complications including skin lesions, ear infections, and tumors (Flurkey et al., 2007). Therefore we chose to conduct our experiments in mice of 18-22 months of age in an attempt to model epileptogenic processes in aging while minimizing the health-related complications that are common in aged mice. We refer to mice of 2–3 and 18–22 months of age as “young” and “aging” respectively for simplicity.

All mice were housed in a vivarium that was maintained between 22–23 °C with a 12-h light on/off cycle (light-on stating at 6:00 am). Food and water were available ad libitum. Hippocampal electrical stimulations and EEG/video recordings were conducted between 10 am and 5 pm. All experimental procedures described below were reviewed and approved by the Animal Care Committee of the University Health Network, in accordance with the Guidelines of the Canadian Council on Animal Care.

### Electrode implantation

Surgeries were similarly performed as previously described (Wu et al., 2008, Jeffrey et al., 2014). The animal was anaesthetized with isofluorane and placed in a stereotaxic frame. After a skin incision to expose the skull surface, three small holes (∼0.5 mm in diameter) were drilled through the skull. Electrodes were inserted into the brain using micromanipulators and then glued onto the skull (Wu et al., 2008). Twisted bipolar electrodes (tips of ∼100 μm apart) were placed into bilateral hippocampal CA3 areas (bregma: −2.5 mm, lateral 3.0 mm and depth 2.5 mm; Franklin and Paxinos, 1997). A reference electrode was positioned at a frontal area (bregma +1.0 mm, lateral 1.0 mm and depth 0.5 mm). All electrodes were made of polyamide-insulated stainless steel wires (outer diameter 200 μm; Plastics One, Ranoake, VA, USA). Implanted animals were allowed to recover for ≥1 week prior to further experimentation. The locations of implanted electrodes were verified by behavioral state-dependent hippocampal EEG activities and/or by later histological assessments.

### Hippocampal CA3 kindling

The animal was placed in a large bowl-shaped glass container for video/EEG monitoring. Unilateral CA3 kindling was conducted using a standard protocol (Albright and Burnham, 1980, Reddy and Rogawski, 2010, Jeffrey et al., 2014). Constant current pulses with monophasic square waveforms, pulse duration of 0.5 ms and current intensities of 10–100 μA were generated by a Grass stimulator and delivered through an isolation unit (model S88, Grass Medical Instruments, Warwick RI, USA). Initially, an ascending series was performed to determine afterdischarges (AD) threshold for individual animals. In the ascending series, a train of current pulses (60 Hz for 2 s) with incremental intensities (10 μA per step) were applied every 30 minutes. The lowest stimulation intensity by which an AD event of ≥5 s was elicited was considered the AD threshold. The animal was then stimulated at 125% of the AD threshold daily for several weeks. The animal was considered kindled when stage 5 motor seizures (see below) were elicited over three consecutive days (Fanelli and McNamara, 1986, Reddy et al., 2010, Jeffrey et al., 2014). An ascending series was similarly conducted in each kindled animal.

### EEG recordings and data analysis

Local differential recordings via twisted bipolar electrodes were used in most of experiments as this recording mode detects signal differences between adjacent electrode tips hence being more effective than mono-polar recordings in sampling local signals and reducing artifacts (Jeffrey et al., 2014, Wu et al., 2015). Mono-polar recordings that detect signal differences between recording and reference electrodes were used if local differential recordings were unsuccessful. EEG signals were collected using a two-channel microelectrode AC amplifier (model 1800, A-M systems, Carlsborg, WA, USA). The input frequency band of the amplifier was set in the range of 0.1–1000 Hz and amplification gain at 1000x. The output signals of the amplifier were digitized at 5000 Hz (Digidata 1440A, Molecular Devices; Sunnyvale, CA, USA). Data acquisition, storage and analyses were done using PClamp software (Version 10; Molecular Devices). In some experiments, a single-channel microelectrode AC amplifier (model 3000, A-M systems) was used to capture ipsilateral (in reference to the unilateral kindling site) AD and TTL-gated switches were used to switch between recording and stimulating modes. When the input frequency band of this amplifier was set in the range of 10–1000 Hz, switching artifacts were usually ≤3 s which masked the early component of evoked AD.

Evoked AD events were recognized as repetitive single spike and poly-spike events with large amplitudes and durations of ≥5 s. AD durations were determined from the end of kindling stimulation to the time point at which AD signals were less than 4 times of standard deviation of pre-stimulation background signals. If needed, original signals were treated with a band filter (1–500 Hz, Bessel) to diminish slow drifts and artifact contaminations prior to the measurements. Contralateral (in reference to the unilateral kindling site) AD events were measured for all animals, and ipsilateral AD were captured and measured from some animals. The contralateral AD durations that were required to reach the first stage 2–5 motor seizure in individual animals were measured and summed, and data were presented as cumulative AD durations to seizure stage (Peterson et al., 1981, Löscher et al., 1998).

Spontaneous or non-evoked hippocampal EEG signals were recorded from individual animals during baseline monitoring and after observation of five consecutive stage 4–5 motor seizures. Large irregular activities that occurred during immobile/sleep behaviors (Buzsáki et al., 2003) and type-2 theta rhythm that corresponded to periods of wake immobility (Sainsbury, 1998) were analyzed. Spectral analysis was used to determine the main frequencies of these activities. Spectral plots (rectangular function, 50% window overlap and spectral resolution 0.3 Hz, PClamp software) were generated from 60-s or 15-s data segments that encompassed the large irregular activities and type-2 theta rhythm. Three spectral plots were averaged for baseline or post-kindling measures in individual animals.

Incidences of aberrant hippocampal EEG spikes were measured from accumulative 20–80 min EEG segments corresponding to immobility/sleep behaviors but excluding the periods with the type-2 theta rhythm (Leung, 1990, Lang et al., 2014). These spikes were recognized with large peak amplitudes (≥8 times of standard deviation of background signals), simple/complex waveforms and durations of 30–150 ms. The event detection function (threshold search method) of PClamp software was used to automatically detect spikes, and detected events were then visually inspected and false events were rejected (El-Hayek et al., 2013, Lang et al., 2014). Spike incidences were determined from the kindled animals that exhibited stage 4 or 5 seizures over 5 consecutive days. To minimize influences of evoked seizures on spike activity, data collected 18–24 hours after the last event of the consecutively evoked seizures were analyzed.

### Motor seizure assessments

Severities of evoked motor seizures were scored using a modified Racine stage (1972) for mice (Reddy and Rogawski, 2010, Reddy et al., 2010). Briefly, stage 0 – no response; stage 1 – behavioral arrest; stage 2 – chewing and head nodding; stage 3 – single or bilateral forelimb clonus; stage 4 − bilateral forelimb clonus and rearing; stage 5 – rearing and falling with forelimb clonus. Motor activity following kindling stimulation was captured by two webcams (Logitech C615) at different angles. Video data were analyzed independently by five investigators whom were blinded to animal groups and kindling information, and averaged daily scores were presented for individual animals. Concordance rates, defined as equal scores by three to five investigators, were 97.6 ± 7.1% for stages 3–5 seizures and 56.8 ± 4.1% for stages 0–2 seizures (p < 0.001), suggesting relatively low individual variability/bias in assessing stages 3–5 seizures hence revealing potential age differences in development of these seizures.

### Brain histological assessments

Brain histological assessments were performed as previously described (El-Hayek et al., 2011, Jeffrey et al., 2014, Wu et al., 2015). Animals were anesthetized via sodium pentobarbital (100 mg/kg, intra-peritoneal injection) and then transcardiacally perfused with 10% neutral buffered formalin solution. The brain was removed and further fixed in 10% formalin with 20% sucrose. Cryostat coronal sections of 50 μm thick were obtained and stained with cresyl violet. A Leica DMRN upright microscope equipped for image tiling and stitching functions was used for image acquisition. Images were analyzed using Image J software (National Institute of Health, USA).

### Statistical analysis

Statistical tests were conducted using Sigmaplot software (Systat Software Inc., San Jose, California, USA). A Student’s t-test or Mann-Whitney Rank Sum Test was used for two-group comparisons. A Chi-square test was used for rate comparisons. Data are presented as means and standard error of the mean (SEM) throughout the text and figures. Statistical significance was set at p < 0.05.

## Results

Of 24 aging mice and 25 young mice used in the present experiments, 17 aging mice and 20 young mice reached a “kindled” status (see below) following daily hippocampal stimulation for up to 5 weeks; kindling experiments were unsuccessful in the remaining animals due to contamination/malfunction of implanted electrodes or complications of skin lesions or infections. The latter cohort of animals was excluded from data analysis.

The intensities of kindling stimulation were comparable between the young and aging mice as there was no significant group difference in initial AD thresholds (46.8 ± 8.1 μA and 41.8 ± 4.9 μA for the young and aging mice; p = 1.0) or AD thresholds assessed in kindled animals (28.8 ± 5.5 μA and 24.3 ± 4.2 μA; p = 0.93). The kindled AD thresholds were significantly decreased from the initial values in both age groups (p = 0.047 and p = 0.007), implicating a state of hippocampal hyperexcitability in the kindled young and aging mice.

### Evoked motor seizures

**Fig. 1:**
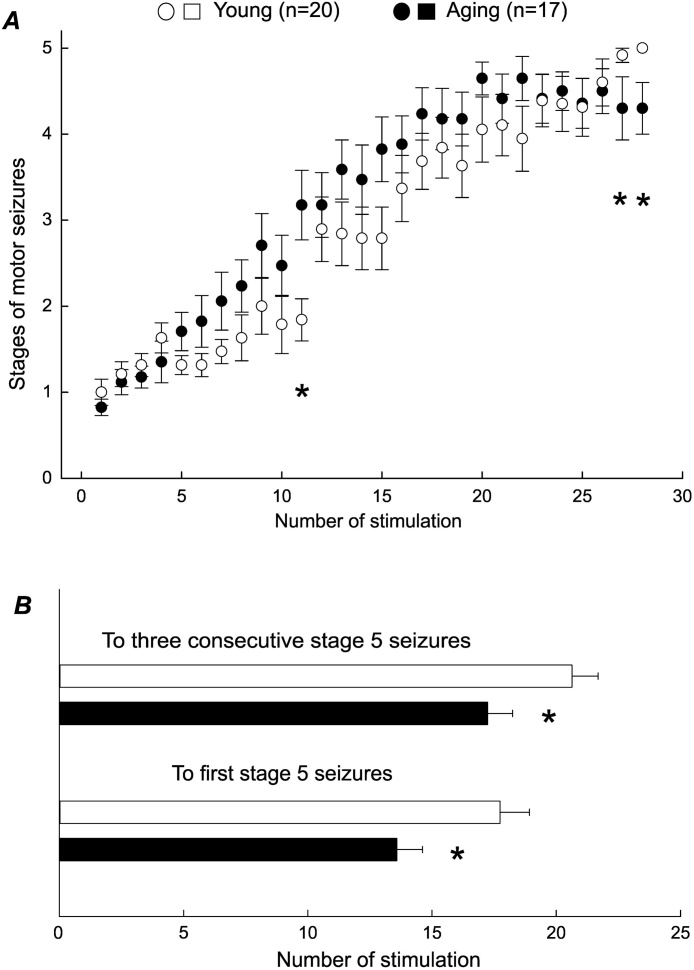
Motor seizure progression in the aging and young mice. A, plots of motor seizure stages vs. numbers of hippocampal stimulation were generated for young and aging mice. B, numbers of hippocampal stimuli to evoke the first stage 5 seizures (bottom) or stage 5 seizures over three consecutive days (top) were determined in individual animals. Here and in subsequent figures, only the stimulations that evoked AD and corresponding responses were analyzed; where the stimulations that failed to evoke AD were excluded from data analysis. Mean and SEM were presented for all data points and significant differences were denoted by * (p < 0.05, Student’s t-test or Mann-Whitney Rank Sum Test) in this and subsequent figures.

The severity of evoked motor seizures were determined using the five stages of the Racine scale (1972) modified for mice (Reddy and Rogawski, 2010, Reddy et al., 2010). Examples of stage 5 motor seizures evoked from a young mouse and an aging mouse are shown in Supplementary Video 1 and 2. We plotted the mean seizure scores vs. the number of hippocampal stimulation to reveal a general trend of motor seizure progression in the young and aging mice. Here and in subsequent text, only the stimulations that evoked AD and corresponding responses were analyzed; the stimulations that failed to evoke AD were excluded from data analysis. As shown in Fig. 1A, the mean seizure scores increased gradually with the number of stimulations in both age groups, and there were no significant group differences in the majority of these mean seizure scores. However, a trend of age difference was noted in the late phase of kindling process as the mean seizure scores approached a plateau after the 20th stimulation in the aging mice but continued an increasing trend to the 28th stimulation in the young mice.

Analyses of the numbers of hippocampal stimulations required to evoke stage 5 motor seizures in individual animals revealed significant age differences. The first stage 5 seizure was observed following 13.6 ± 1.0 hippocampal stimulation in the aging mice but not until 17.7 ± 1.2 stimulation in the young mice (p = 0.013; Fig. 1B). The aging mice were kindled, defined as stage 5 seizures evoked over three consecutive days (Fanelli and McNamara, 1986, Reddy et al., 2010, Jeffrey et al., 2014), following an average of 17.2 ± 1.0 hippocampal stimulations; whereas the young mice were kindled after 20.6 ± 1.1 stimulations (p = 0.027; Fig. 1B).

Vigorous jumps following kindling stimulation were more frequently observed in the young mice than the ageing mice. An example of such jump behavior in a young mouse is shown in Supplementary Video 1. Specifically, 19 of the 20 young mice exhibited jumps and individual animals jumped 4-12 times after a stage 4 or stage 5 seizure; whereas only 5 of the 17 aging mice jumped, with 2-4 jumps following a stage 5 seizure. The proportion of animals that exhibited the jump behavior was significantly greater in the young group than the aging (p < 0.001). It is presently unclear whether these jumps share common features with those previously observed from rats following extended amygdala or perforant path kindling (Pinel and Rovner, 1978, Michalakis et al., 1998, Brandt et al., 2004). We speculate that these jumps likely represent a type of irritated motor response as they were not concurrent with other severe convulsive behaviors (such as barrel rotations and/or wild running) in comparison to jumping seizures observed in mouse models of brain ischemia (El-Hayek et al., 2011, Wang et al., 2015, Wu et al., 2015).

### Evoked hippocampal AD

**Fig. 2:**
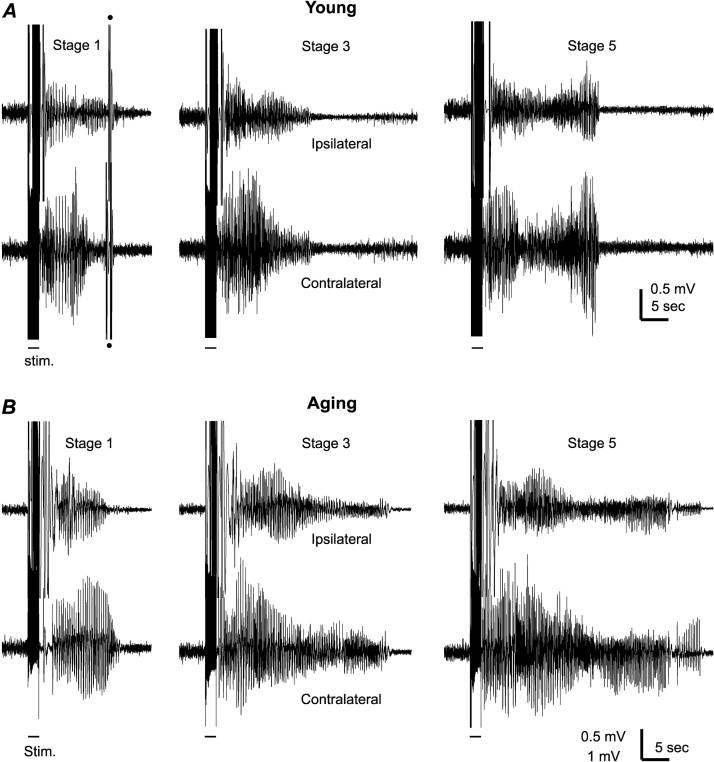
Examples of evoked hippocampal AD. Bilateral hippocampal AD corresponding to stage 1, 3 and 5 motor seizures were collected from a young mouse (A) and an aging mouse (B). The times of applied stimulation (60 Hz for 2 s) were indicated by short bars below traces. ‘Ipsilateral’ and “contralateral” were in reference to the kindling stimulation site. Artifacts were marked with filled circles. Post-stimulation artifacts in ipsilateral traces were due to switches between stimulating and recording modes in the amplifier used (see Methods).

Examples of hippocampal AD collected from a young mouse and aging mouse are shown in Fig. 2, where ipsilateral and contralateral (in reference to the stimulation site) AD corresponding to stage-1, stage-3 and stage-5 motor seizures are illustrated. The durations of hippocampal AD were measured using the contralateral electrode to avoid complications of stimulation artifacts in ipsilateral responses. These measures may closely resemble ipsilateral AD durations as the rodent hippocampus has strong bilateral interconnection (Amaral and Witter, 1989) and coherent bilateral hippocampal AD were observed from some animals in the present experiments (Fig. 2A–B).

**Fig. 3:**
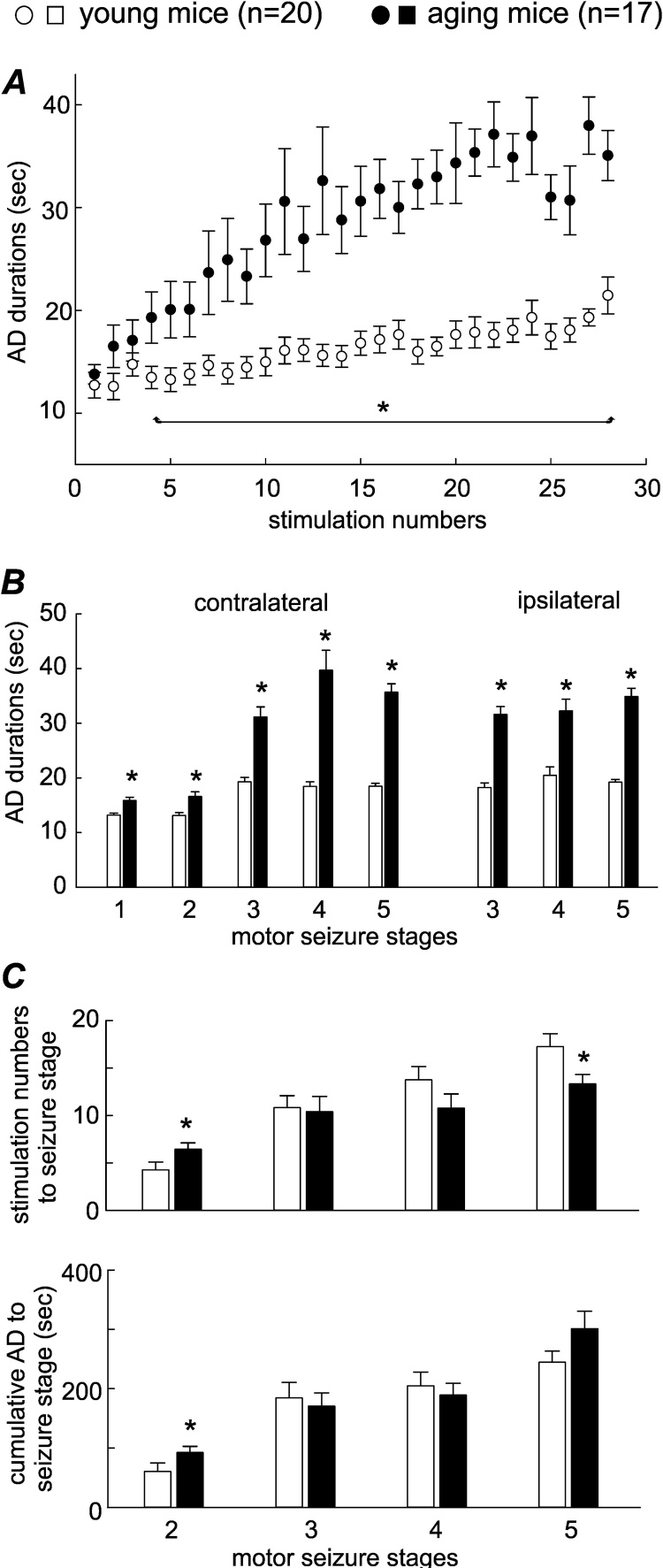
Hippocampal AD measures. A, mean values of contralateral AD durations vs. the number of hippocampal stimulation were plotted for two age groups. B, contralateral (left) or ipsilateral (right) AD corresponding to different stages of motor seizures were measured from individual animals and AD durations were grouped for comparison between the young and aging mice. Contralateral AD corresponding to stage 1–5 seizures were measured from 124, 48, 31, 30 and 71 events in the aging mice and from 159, 100, 26, 28 and 65 events in the young mice respectively. Ipsilateral AD corresponding to stage 3–5 seizures were measured from 33, 3 and 55 events in the aging mice and from 19, 53 and 44 events in the young mice. C, the numbers of stimulations and cumulative AD durations to reach stage 2–5 seizures were measured from individual animals, and data were grouped for comparisons between the young and aging mice. Cumulative AD durations to stage 2–5 seizure were measured from 14, 14, 9 and 17 events in the aging mice and from 19, 11, 14 and 20 events in the young mice respectively.

The mean durations of contralateral AD were plotted vs. the number of hippocampal stimulations to reveal a general trend of increasing hippocampal activity in the young and aging mice (Fig. 3A). Mean AD durations corresponding to the first four stimulations were not significantly different between the aging and young mice. Age differences with longer AD in the aging mice became significant following subsequent stimulations (p = 0.033-p ≤ 0.001). In the late phase of kindling process (after the 20th stimulation), the mean AD durations were about twice as long in the aging mice relative to the young mice.

Significant age differences in AD durations were also observed by analyzing AD in relation to motor seizure stages in individual animals. Contralateral AD corresponding to stage 3–5 seizures were much longer in the aging mice (31.2 ± 1.8, 39.7 ± 3.6 and 35.7 ± 0.8 seconds measured from 31, 30 and 71 events respectively) than in the young mice (19.3 ± 0.8, 18.5 ± 0.9 and 18.5 ± 0.5 seconds measured from 26, 28, and 65 events respectively; p < 0.001); whereas contralateral AD corresponding to stage 1–2 seizures were marginally longer in the aging mice (15.9 ± 0.6 and 16.6 ± 0.8 seconds measured from 124 and 48 events) relative to the young mice (13.2 ± 0.4 and 13.1 ± 0.5 seconds measured from 159 and 100 events; p = 0.004 and p < 0.001; Fig. 3B). Ipsilateral AD were recorded from some young and aging animals (n = 10 in each group) using TTL-gated switches between stimulating and recording modes (see Methods). While ipsilateral AD corresponding to stage 1–2 seizures could not be reliably measured due to relatively large switching artifacts and short responses, ipsilateral AD corresponding to stage 3–5 seizures were evidently longer in the aging mice (31.6 ± 1.4, 32.3 ± 2.1 and 34.9 ± 1.5 seconds measured from 33, 3 and 55 events respectively) than in the young mice (18.2 ± 0.9, 20.5 ± 1.6 and 19.2 ± 0.5 seconds measured from 19, 53 and 44 events respectively; p = 0.003-p < 0.001; Fig. 3B).

The numbers of hippocampal stimulations and cumulative AD durations to reach the first event of stage 2-5 seizures (Peterson et al., 1981, Löscher et al., 1998) were analyzed to further explore age differences in kindling process. As shown in Fig. 3C, the aging mice needed slightly more stimulations and longer cumulative AD durations to reach stage 2 seizures relative to the young mice (stimulation numbers 6.4 ± 0.7 vs. 4.3 ± 1.8 and cumulative AD durations of 92.5 ± 10.3 vs. 60.6 ± 14.2 seconds, n = 14 and 19 measurements, p = 0.015 and 0.028). There were no significant age differences in the two measures to reach stage 3-4 seizures (stimulation numbers of 10.4 ± 1.6 and 10.8 ± 1.5 vs. 10.8 ± 1.2 and 13.8 ± 1.4; cumulative AD durations of 170.4 ± 22.4 and 189.1 ± 19.7 vs. 184.6 ± 25.9 and 204 ± 23.3 seconds, n = 14 and 9 measurements in the aging and n = 11 and 14 measurements in the young mice; p > 0.7). Fewer stimuli were needed to reach stage 5 seizures in the aging mice than in the young mice (13.6 ± 1.0 vs. 17.7 ± 1.2, n = 17 and 20 measurements, p = 0.013), but the cumulative AD durations to stage 5 seizures were not significantly different between the two age groups (301.0 ± 29.2 vs. 244.4 ± 18.6 seconds, p = 0.18).

Together the above observations suggest that the total cumulative AD durations required to reach kindled seizures were not substantially altered in the aging mice in comparison to the young mice and that the faster progression to stage 5 seizures in the aging mice may be predominantly due to prolonged AD (see Discussion).

### Spontaneous hippocampal rhythms and aberrant spikes

**Fig. 4:**
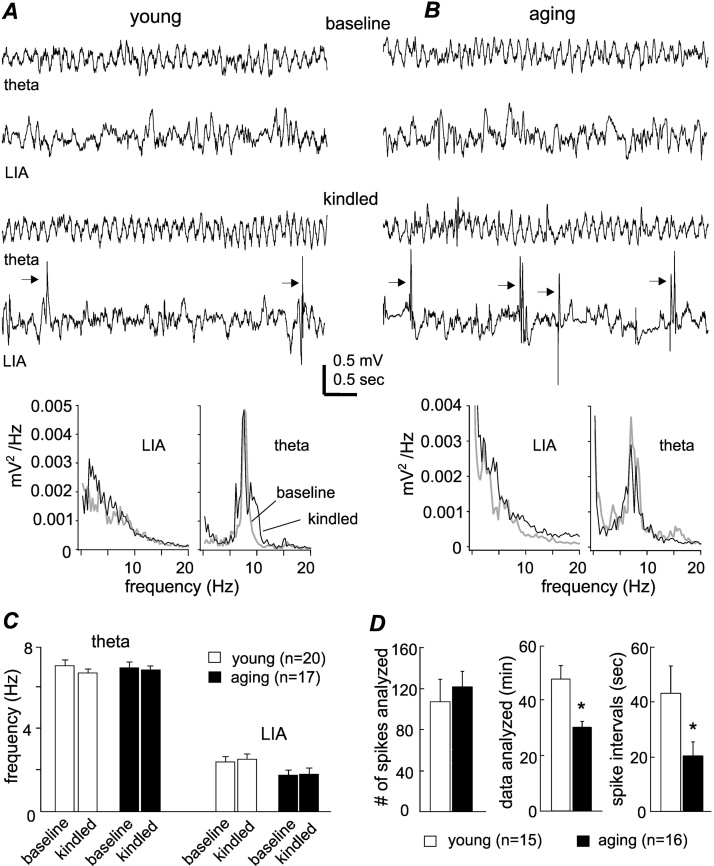
Hippocampal rhythms and aberrant hippocampal spikes. A–B, representative EEG traces were collected from a young mouse and an aging mouse prior to kindling (baseline, top panels) and after observation of stage 4–5 motor seizures over five consecutive days (kindled, bottom panels). The traces showing the type-2 theta rhythm and large irregular activities (LIA) were denoted. Spikes were indicated by arrows. Spectral plots below were generated from 15- or 60-sec data segments including illustrated EEG signals. C, the main frequencies of the LIA and type-2 theta rhythm were measured from individual animals and grouped for comparison between the young and aging mice. D, ipsilateral hippocampal spikes were measured from kindled young and aging mice (n = 15 and 16). Spike detection was performed in EEG segments corresponding immobility/sleep behaviors. The numbers of spikes detected, the durations of EEG segments analyzed and inter-spike intervals measured for the two groups of animals were presented.

Spontaneous or non-evoked hippocampal signals were analyzed to explore whether hippocampal network activities are differentially altered by kindling process in the young and aging mice. The rodent hippocampus is known to exhibit large irregular activities that occur during immobility/sleep behaviors and manifest in the delta frequency band (0.5–4 Hz) (Buzsáki et al., 2003). Rhythmic activities in the theta band (5–12 Hz), called type-2 theta rhythm, occur in wake immobility and are thought to be associated with a light level of arousal/alertness (Sainsbury, 1998). We focused on the large irregular activities and type-2 theta rhythm because their occurrence is independent of active motor behaviors that may potentially differ between the young and aging mice. The large irregular activities and type-2 theta rhythm, with waveforms and frequencies similar to those previously described (Sainsbury, 1998, Buzsáki et al., 2003, El-Hayek et al., 2013), were consistently observed from the two groups of mice (Fig. 4A–B). The main frequencies of these EEG activities were determined via spectral analysis from ipsilateral hippocampal signals. The large irregular activities and type-2 theta rhythm were not significantly altered in frequency following hippocampal CA3 kindling in both age groups. There was no significant age difference in these rhythms, although a trend of lower frequencies of the large irregular activities was noted in the aging mice relative to the young mice (p = 0.057; Fig. 4C).

Aberrant hippocampal EEG spikes were observed following hippocampal CA3 kindling but not during baseline monitoring in the young and aging mice (Fig. 4A–B). In keeping with previous studies (Leung, 1990, Lang et al., 2014), these spikes displayed large amplitudes and single or complex waveforms; the spikes also occurred during immobility and sleep behaviors but not during active movement, exploration behaviors, or in periods exhibiting the type-2 theta rhythm. To determine spike incidences in kindled animals and to minimize influences of evoked seizures on spike activity, EEG data collected 18–24 h after the last event of 5 consecutive stage 4-5 motor seizures were analyzed in individual animals. Our focus was on ipsilateral spikes as they were more frequent and robust than contralateral events. Overall, ipsilateral spike incidences were greater in the aging mice relative to the young mice, as the aging mice displayed similar numbers of spikes in shorter data segments and had briefer inter-spike intervals (Fig. 4D).

### Brain histological observations

**Fig. 5:**
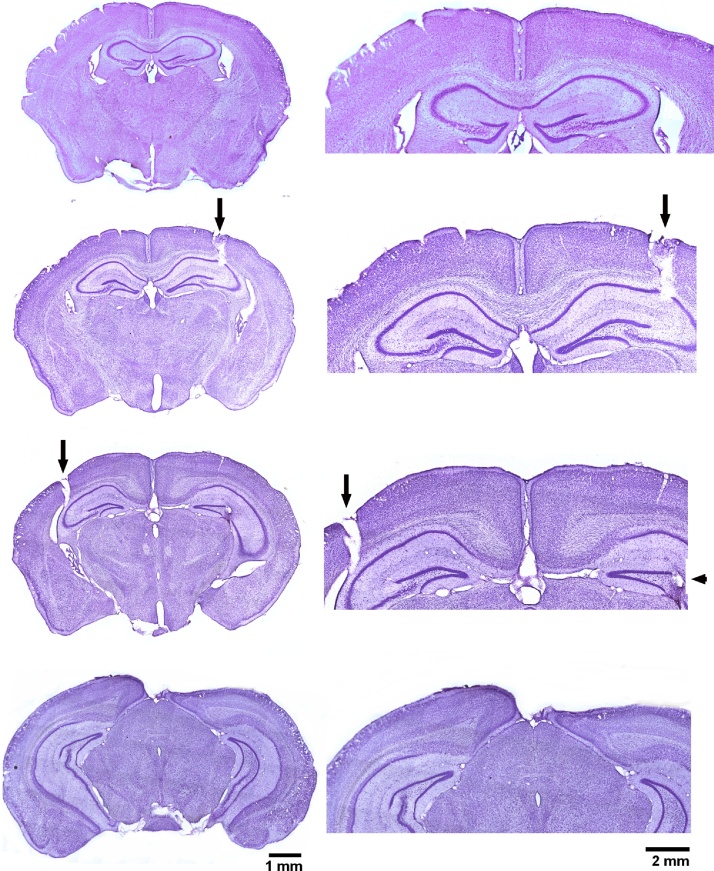
Representative images of brain sections obtained from a kindled aging mouse. Brain histological process was performed after observation of evoked stage 4–5 seizures over 5 consecutive days. Cryostat coronal sections of 50 μm thick were stained with cresyl violet, and representative images were obtained at four coronal levels. Parts of enlarged images are presented at right. Vertical arrows denote the track of implanted electrodes, and a horizontal arrow indicates the location of electrode tip. Note the absence of gross brain lesion in the kindled aging mouse.

**Fig. 6:**
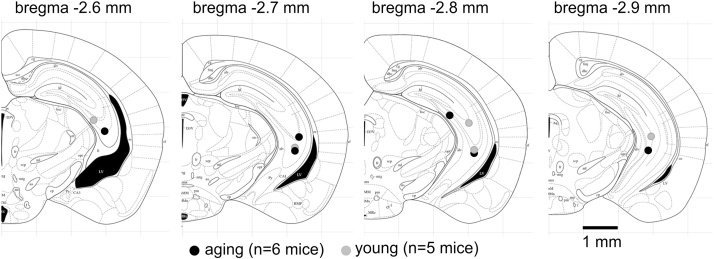
Schematic diagrams showing the locations of recognized electrode tips. Coronal sections of mouse brain at bregma −2.6 mm, −2.7 mm, −2.8 mm and −2.9 mm are schematically presented in diagrams. Locations of electrode tips recognized in kindled mice (n = 6 or 5 for aging or young mice) are denoted by filled grey and black circles respectively.

We performed brain histological assessments to verify the location of implanted electrodes and to examine potential gross brain injury in kindled animals. Seven young mice and 10 aging mice were euthanized for histological assessments after observation of stage 4–5 motor seizures over five consecutive days. Coronal brain sections of 50 μm thick were stained with cresyl violet for general morphological analyses. Representative images of brain sections from a kindled aging mouse are shown in Fig. 5. The tracks of implanted hippocampal electrodes were recognized in all 17 animals examined, and their locations were in hippocampal areas (bregma −2.5–2.9 mm and lateral 2.5–3.5 mm) appropriate to designated stereotaxic coordinates. The locations of electrode tips recognized in kindled mice (n = 6 or 5 for aging or young mice) were schematically presented in Fig. 6. There were no evident gross lesions such as structural deformation, cavity or dark-stained scar tissues in hippocampal and other brain areas in all mice examined. The areas of dorsal, middle and ventral hippocampal tissues were measured in brain sections corresponding to three coronal levels (bregma −1.9 to −2.0 mm, bregma −2.5 to −2.6 mm and bregma −3.0 to −3.1 mm). There were no significant age differences in these measurements (p ≥ 0.1935), with dorsal, middle and ventral hippocampal areas of 123.4 ± 3.3, 301.7 ± 11.6 and 395.9 ± 17.7 square pixels in the aging mice (n = 10) and 124.4 ± 5.5, 266.5 ± 21.0 and 353.9 ± 18.6 square pixels in the young mice (n = 7) respectively.

While brain histological examinations were performed in a portion of kindled mice, hippocampal EEG rhythms with similar waveforms and frequencies were consistently observed in the aging and young mice before and following kindling stimulation (see above). The latter provided electrographic evidence verifying electrode implantation and general functionality of targeted hippocampal CA3 areas. Together these histological and EEG observations suggest similar electrode implantations but no gross brain lesion in the aging and young mice examined.

## Discussion

The goal of our present experiments is to explore whether hippocampal CA3 kindling seizures are different between the aging and young C57 black mice. We observed longer hippocampal AD, faster progression to stage 5 seizures and more frequent spikes in the aging mice relative to the young mice. These age differences were unlikely to be due to variations in hippocampal electrode implantation and stimulation parameters because AD thresholds and hippocampal EEG rhythms measured before and following kindling were comparable between the two groups of mice and hippocampal electrode tracks were recognized histologically in all animals examined. Collectively, these observations are suggestive of a trend that the overall susceptibility to hippocampal CA3 kindling seizures is increased in the aging mice.

### Comparison with previous kindling studies in aged/middle-aged and young rats

Previous studies have compared kindling seizures between aged and young rats (26–28 and 3 months of age respectively; de Toledo-Morrell et al., 1984) or between middle-aged rats (12–15 months of age) and young rats (Fanelli and McNamara, 1986). These studies demonstrate that progression to stage 5 seizures following perforant path kindling is slower in the aged or middle-aged rats relative to the young rats. These age differences are thought to be due at least partly to a loss of glutamate synapses or increased GABAa/benzodiazepine binding in the dentate gyrus of aged or middle-aged rats (de Toledo-Morrell et al., 1984, Fanelli and McNamara, 1986, Geinisman et al., 1978, Geinisman et al., 1986, Gray and Barnes, 2015). These findings appear to be contradictory to the trend we observed in the mouse model with respect to age influences on kindling seizure progression.

However, the kindling sites (perforant path vs. hippocampal CA3) differ between the previous studies and our present experiments. Although it is currently unclear whether the dentate gyrus of aging C57 mice undergoes similar alterations as the aged/middle-aged rats, regional AD and motor seizure development following the perforant path or CA3 kindling are likely different. In addition, species differences are known to be a profound influencing factor in animal models of seizures/epilepsy (Grone and Baraban, 2015). Relative to rats, mice do not exhibit somatostatin-immunoreactive signals in the molecular layer of the dentate gyrus (Buckmaster et al., 1994), have fewer number and slower maturation of adult-born hippocampal neurons (Snyder et al., 2009), and express lower levels of the I_h_ channel in hippocampal pyramidal neurons (Routh et al., 2009). These differences may affect kindling seizure development as hippocampal neurogenesis is enhanced by kindling epileptogenesis (Parent, 2007) and somatostatin-positive GABAergic interneurons and the I_h_ are involved in regulation of hippocampal neuronal activities. Furthermore, there are strain differences in kindling seizures (Löscher et al., 1998, Racine et al., 1999, McIntyre et al., 1999) or other seizure models (Schauwecker and Steward, 1997, McKhann et al., 2003, McLin and Steward, 2006, Schauwecker, 2012, Klein et al., 2015). The rat strains used in the previous studies (Fischer 344 and Sprague-Dawley; de Toledo-Morrell et al., 1984, Fanelli and McNamara, 1986) and the mouse strain (C57BL/6N) used in our present experiments are different in lifespans and aging processes (Flurkey et al., 2007, Harrison et al., 2009, Sengupta, 2011, Dutta and Sengupta, 2016). Therefore, the differences in kindling sites, animal species and other factors yet to be disclosed may attribute to the apparent discrepancy in kindling seizure developments between the previous studies (de Toledo-Morrell et al., 1984, Fanelli and McNamara, 1986) and our present experiments. Future studies that stimulate different sites in commonly used mouse strains may validate the influences of aging on kindling seizures in mouse models.

### Assessments of kindling seizure progression in our model

Previous studies have indicated cumulative AD durations as the principal factor in the acquisition of kindled seizures (Peterson et al., 1981, Löscher et al., 1998). Specifically, the total cortical AD that must be elicited to produce kindling using once daily stimulation paradigm is in the range of 200–300 s (Peterson et al., 1981). In several commonly used rat strains that undergo daily amygdala kindling, the mean values of cumulative amygdala AD durations to reach stage 5 seizures are in a range of 126–334 s (Löscher et al., 1998). In our present experiments, the mean values of cumulative hippocampal AD durations estimated to reach stage 5 seizures were 244 and 301 s for the young and aging mice, which are in keeping with the previous studies in rat models (Peterson et al., 1981, Löscher et al., 1998). As the cumulative AD durations to reach stage 3–5 seizures were largely comparable in the aging and young mice (Fig. 3C), our data further support the concept that “a relatively constant total of AD must be accumulated before kindled seizures will be elicited” (Peterson et al., 1981, Löscher et al., 1998).

The number of stimulations required to evoke stage 5 seizures is commonly used to assess kindling seizure progression or kindling rate under various experimental conditions (Peterson et al., 1981, de Toledo-Morrell et al., 1984, Fanelli and McNamara, 1986, Löscher et al., 1998). In our experiments, fewer stimuli were needed to evoke the first and three consecutive stage 5 seizures in the aging mice relative to the young mice. These observations, taken together with the lack of significant age difference in the cumulative AD durations to stage 5 seizures, are suggestive of overall faster kindling process in the aging mice. However, progression to other seizure stages was either comparable or slower in the aging mice, as there were no significant age differences in the stimulation numbers and cumulative AD durations to stage 3–4 seizures and slightly more stimuli and longer cumulative AD were needed in the aging mice to reach stage 2 seizures. Considering that AD corresponding to stage 3–5 seizures were much longer in the aging mice than in the young mice, we postulate that these prolonged AD may largely attribute to the overall faster progression to stage 5 seizures in the aging group.

### Mechanisms that may underlie prolonged hippocampal AD in the aging mice

Previous studies have shown that the CA3 circuitry is altered towards hyperactivity/hyperexcitability in aging/aged animals. For example, hippocampal CA3 place cells (a group of CA3 pyramidal neurons that show specific spiking patterns during spatial memory tasks) display higher firing rates but fewer plasticity changes in memory-impaired aged rats compared to young rats (Wilson et al., 2005). When examined in hippocampal slices in vitro, CA3 population rhythms and underlying GABAergic activities are attenuated or altered in aged animals (Vreugdenhil and Toescu, 2005, Lu et al., 2011, Kanak et al., 2013, Villanueva-Castillo et al., 2017); whereas induced CA3 epileptiform field potentials and burst firings of CA3 pyramidal neurons (Patrylo et al., 2007, Kanak et al., 2011, Simkin et al., 2015) are enhanced in aged animals relative to young animals. Our lab has previously examined CA3 neuronal activities of young and aged C57 black mice in vivo and in vitro (El-Hayek et al., 2013, Moradi-Chameh et al., 2014). Our data show that intermittent hippocampal EEG spikes are recognizable in aged mice but not in young mice; CA3 epileptiform activities, induced by pharmacological dis-inhibition or high frequency afferent stimulation in hippocampal slices, are more frequent and robust in aged mice than in young mice; CA3 pyramidal neurons of aged mice are featured with more positive resting potentials, higher propensity of burst firing and attenuated rhythmic IPSPs relative to CA3 pyramidal neurons of young mice. Based on these findings, we postulate that kindling stimulation may exacerbate the “normal” CA3 hyperactivity/hyperexcitability that occurs during aging and thus play an important role in prolonging AD in the aging mice.

Previous studies have demonstrated that neuronal injury and/or structural alterations in hippocampal and adjacent areas can occur in rat kindling models. For example, kindling by stimulating the hippocampus, perforant path, or amygdala, has been shown to cause apoptosis and proliferation in dentate gyrus neurons (Bengzon et al., 1997), apoptosis or necrosis of hippocampal neurons and neurons in other areas (Pretel et al., 1997), neuronal losses in hippocampal, entorhinal and rostral endopyriform nucleus areas (Cavazos et al., 1994), structural alterations in the dentate gyrus (Danzer et al., 2010, Singh et al., 2013), alterations and remodeling of piriform cortical GABAergic interneurons (Pollock et al., 2014) and pro-inflammatory process-related hippocampal neuronal loss (Tan et al., 2015). Although C57 black mice are less vulnerable than other mouse stains to kainate-induced hippocampal injury and seizure activities (Schauwecker and Steward, 1997, McKhann et al., 2003, McLin and Steward, 2006) and we did not observe gross brain lesions in the kindled C57 mice, it is very likely that the hippocampal injury/alterations following kindling seizures as previously demonstrated in rat models occur with varied degrees in our model. In addition, implantations of intra-hippocampal electrodes in our experiments are likely associated with local gliosis, cell injury and/or inflammatory responses as previously demonstrated in other models (Winslow and Tresco, 2010, Constant et al., 2012, Ravikumar et al., 2014). The injuries and structural alterations resulting from kindling seizures and the local responses related to electrode implantation may be exacerbated in the aging mice hence attributing to prolonged hippocampal AD and faster motor seizure progression.

Other factors, particularly seizure-induced increase in blood-brain barrier permeability (Lamas et al., 2002, Vazana et al., 2016) and associated pro-inflammatory processes and astrocyte-glia dysfunction (Vezzani et al., 2013, Iori et al., 2016, Eyo et al., 2017), may also play important roles. In view of age-dependent blood-brain barrier breakdown in the human hippocampus (Montagne et al., 2015) and decreased claudin-8 expression (a member of tight junction proteins in endothelial cells) in kindled rat hippocampal tissues (Lamas et al., 2002), kindling may enhance the interplay of compromised hippocampal blood-brain barrier and pro-inflammatory signals and facilitate AD prolongation in the aging mice. As the age differences in AD durations became significant after the first few days of kindling in our model (Figs. 3A), it would be of great interest to examine whether young and aging mice differ in CA3 neuronal activity, hippocampal blood-brain barrier permeability and neuronal injury following the first several hippocampal stimulations and whether applications of anti-inflammatory agencies in this period affect AD and motor seizure progression in an age-dependent manner.

### Limitations of our present experiments

There are several limitations or drawbacks in our present experiments. In particular, our histological assessments were limited in revealing brain injury and/or structural alterations at cellular and micro-circuitry levels as previously demonstrated (Bengzon et al., 1997, Cavazos et al., 1994, Danzer et al., 2010, Singh et al., 2013, Pollock et al., 2014, Tan et al., 2015). As such brain injury and/or structural alterations may be exacerbated in the aging mice and account for at least partly for prolonged AD and faster seizure progression, further experiments with detailed brain histological assessments are required to address this critical issue in our model. In addition, our experiments were conducted in male mice in an attempt to avoid the influences of varied sex hormones in female mice. However, female sex hormones are known to have strong influences on seizure/epileptic activity (Velíšková and Desantis, 2013, Scharfman and MacLusky, 2014, Taubøll et al., 2015) particularly hippocampal kindling seizures (Reddy et al., 2010). As female C57 black mice of ≥16 months of age may be in a post-estrous cycle state (Diaz Brinton, 2012) whereas sex hormones in male C57 mice decline slowly in an age range of 8-24 months (Chen et al., 2015), the influences of aging on hippocampal CA3 kindling seizures may substantially differ between female and male mice. Furthermore, caution should be taken when discussing the clinical relevance of our observed hippocampal AD and motor seizures, as these are evoked responses and distinct from the spontaneous recurrent seizures seen in patients with epilepsy or other animal models (Bertram, 2007, Sharma et al., 2007, Lévesque et al., 2016, Löscher, 2017).

## Conclusion

Our experiments provide original data showing that the aging mice exhibit longer AD and faster progression to stage 5 seizures relative to the young mice following hippocampal CA3 kindling. Despite the limitations particularly in detailed assessments of neuronal injury and structural alterations in our model, our present data are suggestive of the view that overall susceptibility to hippocampal CA3 kindling seizures is increased in the aging C57 black mice. Our work together with the previous studies (de Toledo-Morrell et al., 1984, Fanelli and McNamara, 1986, Hattiangady et al., 2011) may promote further experimental investigations regarding the influences of aging on seizure/epilepsy susceptibility.

## Funding and Disclosure

This study is supported by research grants from Natural Science and Engineer Research Council of Canada (RGPIN-2015-04153), Eplink of Ontario Brain Institute and Canadian Institute of Health Research. The authors declare no competing financial interests with any aspect of this work.

## Author contributions

KR Stover, S Lim, TL Zhou, PM Stafford, J Chow H Li, S Mylvaganam and N Sivanenthiran contributed to experimentation and/or data analysis. CP Wu contributed electrode implantation surgery. KR Stover, DF Weaver, JH Eubanks and L Zhang contributed to experimental design, data discussion and manuscript writing.
