# Supplementary material for: Susceptibility to hippocampal kindling seizures is increased in aging C57 black mice
Source: IBRO Rep. 2017 Sep 24;3:33–44. doi: 10.1016/j.ibror.2017.08.001 (PMC6084868; doi:10.1016/j.ibror.2017.08.001)
Supplement: Supplementary file 1 [file mmc1.docx]

**Video legends**

**Supplementary Video 1**: Motor responses of a young mouse in response to the 23^rd^ daily hippocampal stimulation. The animal exhibited a stimulation-induced stage 5 seizure (fall with limb clonus and extension) at approximately the 39^th^ second of video time and repetitive jumps afterwards.

**Supplementary Video 2**: Motor responses of an aging mouse in response to the 18^th^ daily hippocampal stimulation. The animal showed a stimulation-induced stage 5 seizure (fall with limb clonus) at approximately the 61^st^ second of video time but without subsequent jump behavior.
